# Assessing the usefulness of the Little Developmental Coordination Disorder Questionnaire-Chinese in Chinese preschoolers: a sex-and age-specific analysis

**DOI:** 10.3389/fpsyg.2024.1321342

**Published:** 2024-01-30

**Authors:** Jing Hua, Tanya Rihtman, Yongmei Peng, Tianjing Wang, Yuantao Su, Wenchong Du

**Affiliations:** ^1^The Women’s and Children’s Health Care Department of Shanghai First Maternity and Infant Hospital, School of Medicine, Tongji University, Shanghai, China; ^2^Shanghai Key Laboratory of Maternal Fetal Medicine, Shanghai First Maternity and Infant Hospital, School of Medicine, Tongji University, Shanghai, China; ^3^Department of Sport, Health Sciences and Social Work, Oxford Brookes University, Oxford, United Kingdom; ^4^Shanghai Center for Women and Children’s Health, Shanghai, China; ^5^School of Medicine, Tongji University, Shanghai, China; ^6^NTU Psychology, School of Social Sciences, Nottingham Trent University, Nottingham, United Kingdom

**Keywords:** developmental coordination disorder, motor coordination difficulty, early identification, preschoolers, LDCDQ, validity, reliability, Chinese children

## Abstract

**Aim:**

This study evaluated the sex-and age-specific usefulness of the Little Developmental Coordination Disorder Questionnaire-Chinese (LDCDQ-CH) in Chinese preschoolers.

**Method:**

A population-based sample of 51,110 children aged 3–5 years was recruited. Internal reliability, construct validity, concurrent validity with the Ages and Stages Questionnaire-third edition (ASQ-3), and discriminant validity with the Movement Assessment Battery for Children-second edition (MABC-2) were assessed. Age and sex effects on LDCDQ-CH scores were analyzed using ANOVA and *t*-tests.

**Results:**

The LDCDQ-CH exhibited excellent internal consistency and reliability across ages and genders. Confirmatory factor analysis supported the 15-item model’s satisfactory fit. Positive and significant correlations were observed between LDCDQ-CH and ASQ-3 scores, indicating robust concurrent validity. Significant associations were found between LDCDQ-CH and MABC-2 scores. Higher scores were observed in older children and girls, indicating age- and sex-related differences in motor functional performance.

**Conclusion:**

The LDCDQ-CH is a reliable and valid tool to support early identification of motor coordination difficulty in Chinese preschoolers, and guiding interventions. Findings support its use across ages and genders, highlighting its potential in the Chinese context. Age- and sex-specific norms are needed for enhanced clinical applicability.

## Highlights

- The LDCDQ-CH shows good to fair reliability and validity in Chinese preschoolers.
- Age- and sex-specific norms are needed for enhanced clinical applicability.

## Introduction

Developmental coordination disorder (DCD), a lifelong neurodevelopmental disorder, characterized by a pervasive difficulty in acquiring age-appropriate motor skills (Subara-Zukic et al., 2022), exhibits a global prevalence of 5–6% (Harris et al., 2015), with slightly higher rates in China at around 7% (Du et al., 2020). Although it is not recommended to make a formal diagnosis of DCD before the age of 5 years (Blank et al., 2019), infants and toddlers who are later diagnosed with DCD are often delayed in the attainment of early gross and fine motor milestones as compared to typically developing children of the same age (Smits-Engelsman et al., 2015; King-Dowling et al., 2016; Hua et al., 2022). However, by the time a child with DCD receives a formal diagnosis, secondary difficulties have often started to emerge (Zwicker et al., 2018; Rodriguez et al., 2019), when an optimal window for early intervention has been missed (Blank et al., 2019). It is therefore imperative to identify young preschool children who are at risk of a later DCD diagnosis, monitor the development of their functional movements and provide corresponding support and intervention as early as possible (Rihtman et al., 2011).

While numerous tools are available to screen for, or diagnose DCD, these are limited in their applicability to younger children (Schoemaker et al., 2008). This may be due to their length, the need for specific examiner expertise, the inclusion of items which may not apply to children below the age of 5 and/or unsuitable information providers of younger children (e.g., school teachers) (Blank et al., 2019). One such example is the Developmental Coordination Disorder Questionnaire (DCDQ’07), which is a valid and reliable parent questionnaire designed to screen for motor difficulties associated with DCD in children aged 5 to 15 years (Wilson et al., 2009). This ecologically valid tool has been translated and validated for use in numerous countries around the world, and is widely used and recognized by clinicians (Kennedy-Behr et al., 2013; Caravale et al., 2014b). However, the items of the DCDQ’07 are not functionally suited to children below the age of 5 years.

As a downward extension of the DCDQ’07, the Little Developmental Coordination Disorder Questionnaire (LDCDQ) was originally developed in Hebrew to identify motor development and coordination difficulties of young preschoolers aged 3- and 4-years (Rihtman et al., 2011). The original LDCDQ demonstrated sound psychometric properties (Rihtman et al., 2011), and since its initial publication, the LDCDQ has been adapted for use in multiple countries including – among others – the Netherlands (Cantell et al., 2018), South Africa (Venter et al., 2015), France (Jover et al., 2013), and Canada (Wilson et al., 2015). These studies have demonstrated the need for a variety of culture- and context-specific amendments, including adjustment of item phrasing and detail, the inclusion of alternate item examples, age-range extension to include 5-year-old children (where children begin schooling later), and the adoption of alternate sub-scores structures. With these local amendments applied, the adapted versions of the LDCDQ have all demonstrated sound psychometric properties.

Moreover, the psychometric investigation of the LDCDQ in different countries have highlighted a range of avenues for further exploration. For example, findings that the LDCDQ items may be appropriate for children older than 4 years (Cantell et al., 2018; Fu et al., 2022; Hudson and Willoughby, 2022), findings related to discriminant validity of 3-year-olds as compared to 4-year-olds (Venter et al., 2015; Wilson et al., 2015; Cantell et al., 2018), and gender differences found in some studies (Cantell et al., 2018) but not others (Rihtman et al., 2011; Hudson and Willoughby, 2022). Additionally, the age- and sex- related variations in some of aspects of reliability and validity (e.g., the internal consistency, construct and discriminant validity) have not been reported specifically in previous studies. Assessing the sex-and age-specific usefulness LDCDQ holds significant importance for several reasons. Firstly, understanding how functional motor performance varies between sexes can provide valuable insights into potential gender differences in motor coordination and performance. Secondly, considering the age-specific usefulness of the LDCDQ is essential because motor development progresses rapidly during early childhood. By examining how the LDCDQ performs across different age groups as a screening tool, we can identify the most appropriate age range for its application and ensure accurate assessment of motor difficulties at specific developmental stages. Additionally, investigating the age- and sex-related differences in the validity and reliability of the LDCDQ in Chinese children is crucial for establishing reliable local norms and benchmarks that can guide clinicians and researchers in the early identification and intervention of motor impairments.

This study reports on the adaptation and population-based psychometric assessment of the LDCDQ for use in mainland China (LDCDQ-CH). The first aim of the study was to ensure cross-cultural validity through rigorous translation and cross-cultural adaptation of the LDCDQ to generate the LDCDQ-CH. Thereafter, the reliability and validity of the LDCDQ-CH were investigated for use in Chinese preschoolers up to the age of 5 years 11 months, in addition to the original target age and 3 and 4 years. The gender-specific reliability and validity of the LDCDQ-CH were also examined with a large population-based sample, as was the discriminant validity.

## Methods

### Participants

**Figure 1:**
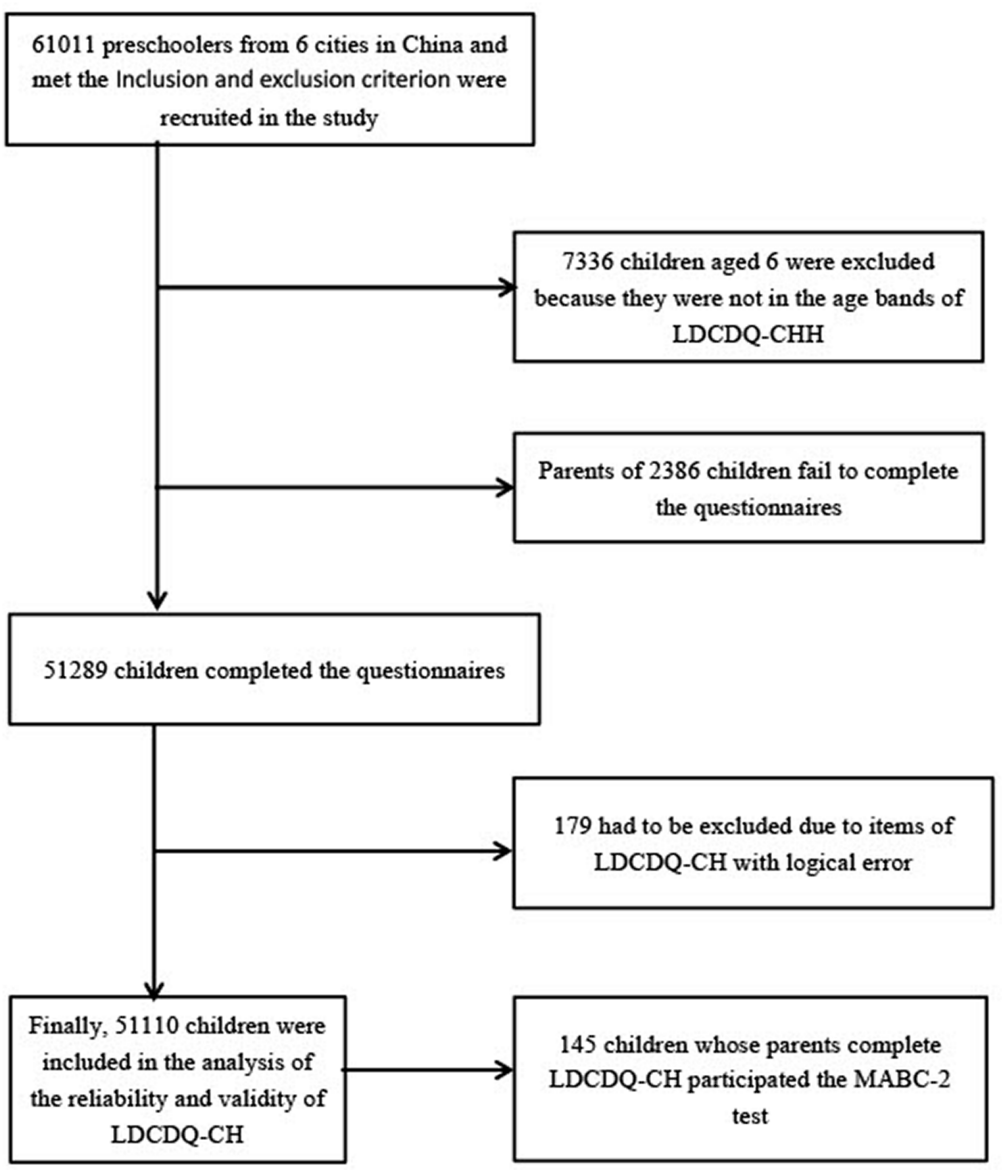
Flow chart of investigated children.

Children/families were recruited from 798 nurseries of 6 provinces (Hainan, Jiangsu, Zhejiang, Anhui, Shanghai, and Fujian) of South-Eastern China, and data collection was undertaken between April–November 2018. Only children aged 3–4 years old from mainstream nurseries were included in the study. Children with severe visual, hearing, or intellectual impairments (as verified by developmental examination records obtained upon nursery enrollment) or other severe developmental disorders who were required to attend special education schools or nurseries according to the local regulations were excluded. Children were also excluded from the study if they had a diagnosis of autism spectrum disorder, severe myopia, amblyopia or other sensory loss or a neuro-motor disorder. Figure 1 provides an overview of the total number of children recruited to the current study (*n* = 61,011), the application of exclusion criteria to generate the LDCDQ-CH study validation sample (*n* = 51,110), and the discriminant validity sub-sample (*n* = 145).

### Measures

*The Little Developmental Coordination Disorder Questionnaire (LDCDQ)* (Rihtman et al., 2011) is a 15-item questionnaire originally designed to screen for motor coordination difficulties in children aged 3 to 4 years, with some researchers extending the age range to 5 years (Cantell et al., 2018; Fu et al., 2022; Hudson and Willoughby, 2022). The questionnaire comprises three sub-categories: control during movement (CDM), fine motor (FM), and general coordination (GC), each consisting of five items. Parents are asked to rate their child’s performance compared to other children of the same age and gender using a Likert scale ranging from 1 (not at all like my child) to 5 (extremely like my child). The maximum score for each sub-category is 25, with a maximum total score of 75. While the LDCDQ does not aim to differentiate between levels of motor competence among children without motor difficulty, lower scores suggest potential motor proficiency difficulties.

*The Ages and Stages Questionnaire-third edition (ASQ-3)* (Squires et al., 2009) was used for the analysis of concurrent validity alongside the LDCDQ. The ASQ-3 is a widely used developmental screening tool that assesses various domains of early childhood development, including communication, gross motor, fine motor, problem-solving, and personal-social skill (Kerstjens et al., 2009). It consists of 30 items and is completed by parents. Each item is scored as ‘yes’ (10 points), ‘sometimes’ (5 points), or ‘not yet’ (0 points), reflecting the child’s ability to perform the behavior. Each domain comprises six questions, with a maximum score of 60. Missing item scores were computed according to the ASQ-3 guidelines, and questionnaires with more than two blank items in a section were excluded from the analysis. The ASQ-3 has been translated and validated for use with Chinese children, with the Chinese version of the ASQ-3 demonstrating fair to good sensitivity and specificity (Wei et al., 2015).

*The Movement Assessment Battery for Children-second edition (MABC-2)* (Henderson et al., 2007) is a diagnostic assessment for the motor impairment of Developmental Coordination Disorder (DCD). In this study, the MABC-2 Age Band 1 (3–6 years old) was used to assess concurrent and discriminant validity. Age Band 1 includes eight tasks categorized into manual dexterity (posting coins, threading beads, drawing trails), aiming and catching (catching and throwing a beanbag onto a mat), and balance (one-leg balance, walking on heels, jumping on mats). Based on the MABC-2 total scores, children were classified into three groups: significant motor impairment (≤ 5th percentile of the total score), at-risk of motor impairment (6–16 percentiles of the total or subtest score), and typical motor performance (>15th percentile of the total or subtests’ score) according to the MABC-2 Examiner’s Manual (Henderson et al., 2007). The MABC-2 has been translated and validated for use with Chinese children and has been found to be a reliable and valid measure for diagnosing motor impairment in this population (Hua et al., 2013).

### Procedure

The study was approved by the Ethics Committee of Shanghai First Maternity and Infant Hospital (KS18156).

*Stage 1:* To ensure the cross-cultural validity (Beaton et al., 2000) of the Chinese LDCDQ (LDCDQ-CH), a rigorous forward-backward translation procedure was employed. The translation process was overseen by a panel comprising three highly experienced pediatricians and two child psychologists, each possessing over 10 years of field expertise. Consistent with the guidelines proposed by Beaton et al. (2000) for cross-cultural adaptation of health questionnaires, the LDCDQ was initially translated into Mandarin. Subsequently, an independent translator performed a back translation, and the original and back-translated versions were meticulously reviewed to ensure conceptual equivalence. The panel also thoroughly assessed the cultural appropriateness of the LDCDQ-CH for Mainland China, ensuring that the phrasing and activities described in the questionnaire items were suitable for the local culture. Notably, to better align with the eating behaviors of Chinese children, the examples of cutlery were expanded to include the use of chopsticks, which are commonly employed in Chinese dining settings (Wong et al., 2002; Li-Tsang et al., 2006).

*Stage 2:* To establish internal consistency and construct validity, in 798 participating nurseries, study notifications were disseminated by class teachers to parents, accompanied by researchers’ contact details for any inquiries. Parents who voluntarily agreed to take part accessed and completed the questionnaire through an online platform. Given the prevalent use of online communication and interactions between parents and their children’s nurseries in China, it was presumed that the parents involved in this study possessed a considerable level of proficiency in completing online questionnaires.

*Stage 3:* To assess concurrent and discriminant validity, children whose parents provided consent for their participation in professional motor testing (MABC-2) were included in further analysis. A total of 145 children were evaluated by clinically qualified and experienced pediatricians who were blinded to the children’s history and LDCDQ-CH questionnaire scores. These pediatricians had more than three years of experience conducting physical examinations on preschool children and were proficient in administering the Chinese MABC-2 tests.

### Data analysis

#### Internal consistency

To obtain an estimate of the internal consistency of the LDCDQ-CH, Cronbach’s alpha and correlated item-total correlation were calculated. We also used “Cronbach’s alpha if item deleted” to analyze if the respective item is consistent with the rest of the scale. The Guttman split-half coefficient was also used to assess the internal validity.

#### Construct validity

To assess the variations in LDCDQ-CH scores across different age groups and between sexes, one-way ANOVA and two-independent-sample *t*-tests were employed, respectively. In the case of ANOVA, post-hoc analysis was conducted using the Least Significant Difference (LSD) test to determine specific differences between groups. Initially, we conducted an exploratory factor analysis (EFA) to investigate the construct of the LDCDQ-CH. The suitability of EFA for our dataset was evaluated using the Kaiser-Meyer-Olkin (KMO) measure of sampling adequacy and Bartlett’s test of sphericity. Subsequently, drawing upon the structure of the LDCDQ (Rihtman et al., 2011), we then employed confirmatory factor analysis (CFA) to assess the construct validity (Wilson et al., 2009). The chi-square value and several fit indices were selected to assess the fit of the CFA models (Houts and Kassab, 1990; Sockloskie, 1990; Hu and Bentler, 1999) including the Incremental Fit Index (IFI), the Comparative Fit Index (CFI), the Normed fit index (NFI) and the Root Mean Square Error of Approximation (RMSEA). Values exceeding 0.90 for the IFI, the CFI, and the NFI are indicative of acceptable model fits. Additionally, a RMSEA below 0.05 suggests a reasonable approximation error in the population (Jöreskog and Sörbom, 1993).

#### Concurrent validity

Concurrent validity examines the relationship between scores obtained from two instruments used to assess motor development. In this study, we investigated the correlation between scores on the LDCDQ-CH and ASQ-3, considering all participants in the analysis. Additionally, we evaluated the correlation between the LDCDQ-CH and MABC-2, focusing on a sub-sample of children. Pearson correlation analysis was employed to assess the association between the total scores and sub-scaled scores derived from the two scales.

#### Discriminant validity

Discriminant validity holds significant importance for screening tools as it determines the test’s ability to distinguish children with and without motor impairment. In our study, we employed a one-way ANOVA to examine score and sub-scaled score differences among three groups: groups with motor impairment, at-risk motor impairment, and typical performance. Specifically, we evaluated the differences in scores between children with significant motor impairment according to the MABC-2, those at risk of motor impairment, and those with typical motor performance. To further investigate these differences, post-hoc analysis using Fisher’s Least Significant Difference (LSD) was conducted. It is crucial to explore age- and sex-related differences in both concurrent and discriminant validity, as early identification of children at risk of motor impairment is of utmost importance.

#### Stratified analysis of reliability and validity

To examine age- and sex-related differences in the validity and reliability of the LDCDQ-CH in Chinese children, we initially investigated the interaction effects between age and sex. A two-way ANOVA for the score of LDCDQ-CH has been conducted with an interaction model. However, we could not find the interaction term (age*sex) is statistically significant at an alpha level of 0.05 (*p* > 0.05). Consequently, we proceeded to separately conduct the analyses of validity and reliability by age and by sex.

## Results

In the final analysis, a total of 51,110 children were included. The participants had a mean age of 4.08 years, with a standard deviation of 0.76. Among them, 12,849 (25.1%) were 3 years old, 21,192 (41.5%) were 4 years old, and 17,069 (33.4%) were 5 years old. In terms of gender, 27,404 (53.6%) children were boys and 23,706 (46.4%) children were girls. Further details regarding participant characteristics can be found in Table 1.

**Table 1:** The children’s socio-demographic and health characteristics in subjects (*n* = 51,110).

Characteristics	*n* (%)
Children’s age	
3	12,849 (25.14)
4	21,192 (41.46)
5	17,069 (33.40)
Gender	
Male	27,404 (53.62)
Female	23,706 (46.38)
BMI	
≤18	40,526 (79.29)
>18	10,584 (20.71)
Mother’s higher education	
No	25,832 (50.54)
Yes	25,278 (49.46)
Father’s higher education	
No	25,237 (49.38)
Yes	25,873 (50.62)
Family per-capita income of every month (RMB)	
≤3w	8,726 (17.07)
>3w	42,384 (82.93)
Preterm birth (<37 gestational week)	
No	46,073 (90.15)
Yes	5,036 (9.85)

### Internal consistency

The internal consistency and reliability of the LDCDQ-CH were found to be high. The Cronbach’s alpha value for all 15 items combined was 0.939, indicating strong internal reliability. Similarly, the Cronbach’s alpha values for children aged 3, 4, and 5 were 0.935, 0.939, and 0.940, respectively. In terms of gender, the Cronbach’s alpha was 0.939 for boys and 0.941 for girls, indicating good internal reliability across different age groups and genders (Coefficients above 0.75 suggest good reliability).

The Cronbach’s alpha coefficients for deleted items are presented in Table 2. The analysis revealed that removing most of items was less than the total value of 0.939, did not increase the Cronbach’s alpha coefficient. However, the item of ‘Sits upright‘(0.940) was weakly higher than the total value of 0.939.

**Table 2:** The item-deleted Cronbach’s alpha coefficients of LDCDQ-CH by different ages and sex.

Items	Total *n* = 51,110	Age			Sex	
		3 *n* = 12,849	4 *n* = 21,192	5 *n* = 17,069	Male (*n* = 27,404)	Female (*n* = 23,706)
Coordination	0.937	0.932	0.937	0.938	0.936	0.939
Building	0.935	0.931	0.936	0.937	0.935	0.938
Drinks	0.935	0.931	0.935	0.936	0.934	0.937
Move place	0.935	0.931	0.935	0.936	0.934	0.937
Sits upright	0.940	0.935	0.940	0.943	0.940	0.942
Catch	0.934	0.930	0.935	0.936	0.934	0.937
Stickers	0.934	0.930	0.934	0.936	0.933	0.936
Kick	0.933	0.929	0.934	0.935	0.933	0.935
Cutlery	0.935	0.931	0.936	0.937	0.935	0.937
Imitate	0.933	0.929	0.933	0.934	0.932	0.935
Pencil	0.933	0.929	0.934	0.935	0.933	0.935
Throw	0.934	0.929	0.934	0.935	0.933	0.936
Playground equipment	0.935	0.931	0.935	0.936	0.935	0.937
Run	0.934	0.930	0.934	0.935	0.933	0.936
Thread	0.936	0.932	0.936	0.947	0.935	0.938

Furthermore, the Guttman split-half coefficient, assessing the total scale’s internal consistency, was 0.913, 0.917, and 0.917 for children aged 3, 4, and 5, respectively. Among boys, the coefficient was 0.918, and among girls, it was 0.913. These coefficients further affirm the strong internal consistency of the LDCDQ-CH across different age groups and genders.

### Construct validity

The mean scores on the LDCDQ-CH differed by age and gender with statistical significance (each *p* < 0.05). Specifically, the mean scores were 65.90, 67.20, and 68.58 for children aged 3, 4, and 5 years old, respectively. Girls had a mean score of 67.92, while boys had a mean score of 66.83. The standard deviations for these scores were 9.30, 9.00, 8.38, 9.12, and 8.67, respectively. Statistical analysis revealed that the mean scores on the LDCDQ-CH were significantly higher in older children compared to their younger counterparts (*p* < 0.001). Additionally, girls obtained higher mean scores than boys, and this difference was also statistically significant (*p* < 0.001).

Initially, an exploratory factor analysis (EFA) was conducted on the 15 items of the LDCDQ-CH to ascertain the measurement structure of the questionnaire. The Kaiser–Meyer–Olkin (KMO) index exhibited a high value of 0.966, suggesting that factor analysis was appropriate for our data. Additionally, the Bartlett’s test of sphericity, which compares our correlation matrices to an identity matrix, yielded highly significant results (x^2^ = 551684.56, *p* < 0.001). However, analysis of the scree plot for factor extraction indicated a one-factor solution as most suitable, deviating from the original construct of the LDCDQ proposed by Rihtman.

**Figure 2:**
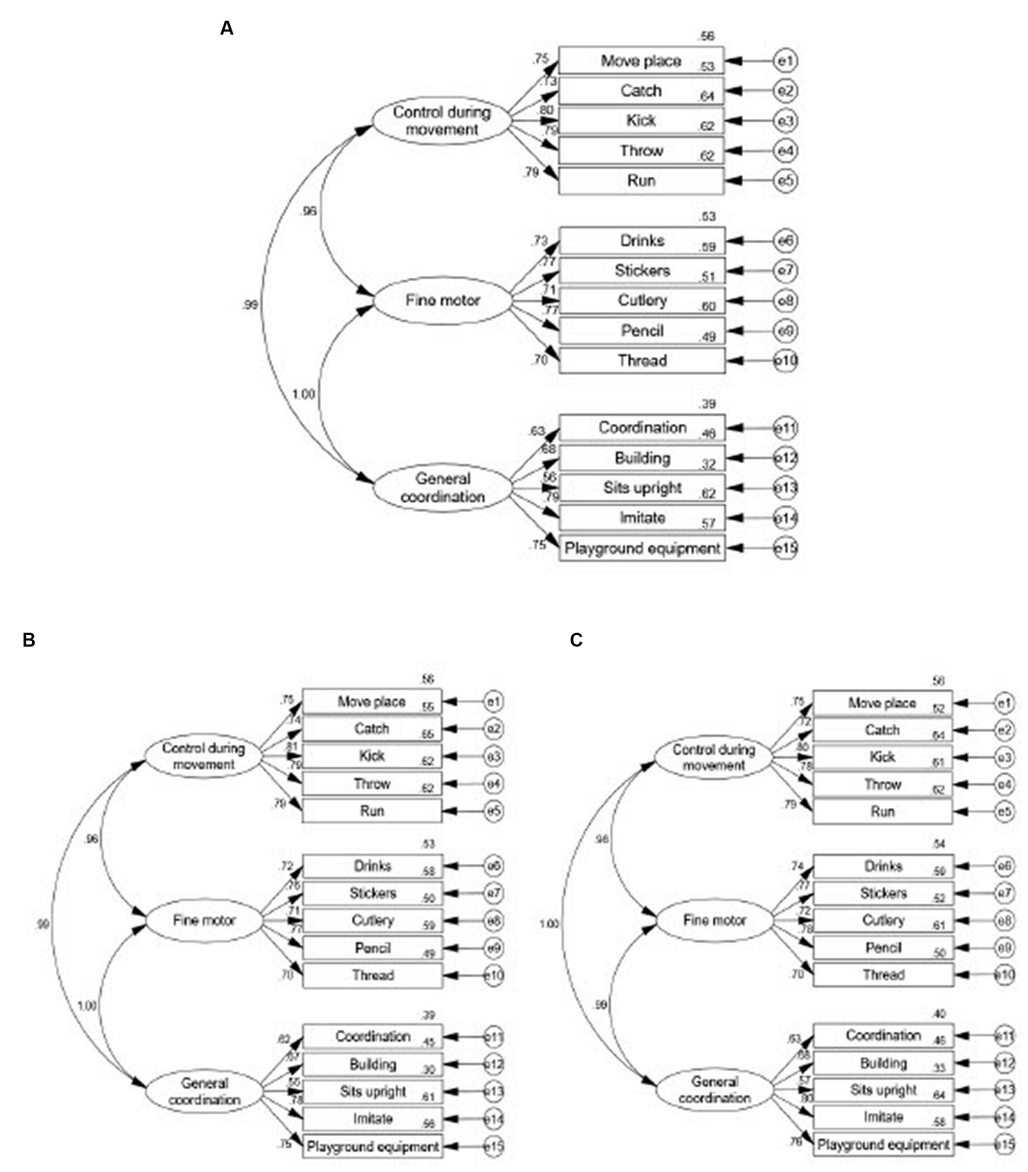
The factor loadings of LDCDQ-CH using confirmatory factor analysis in the total participant population and by sex. **(A)** Factor model in total subjects (*n* = 51,110, x^2^/df = 402.731, *p* < 0.001, NFI = 0.927, CFI = 0.927, IFI = 0.927, RMSEA = 0.089, TLI-0.912). **(B)** Factor model in males aged 3–5 years old (*n* = 27,404, x^2^/df = 219.940, *p* < 0.001, NFI = 0.924, CFI = 0.925, IFI-0.925, RMSEA = 0.089, TLI = 0.909). **(C)** Factor model in females aged 3–5 years old (*n* = 23,706, x^2^/df = 184.528, *p* < 0.001, NFI = 0.929, CFI = 0.929, IFI-0.929, RMSEA = 0.088, TLI = 0.915).

**Figure 3:**
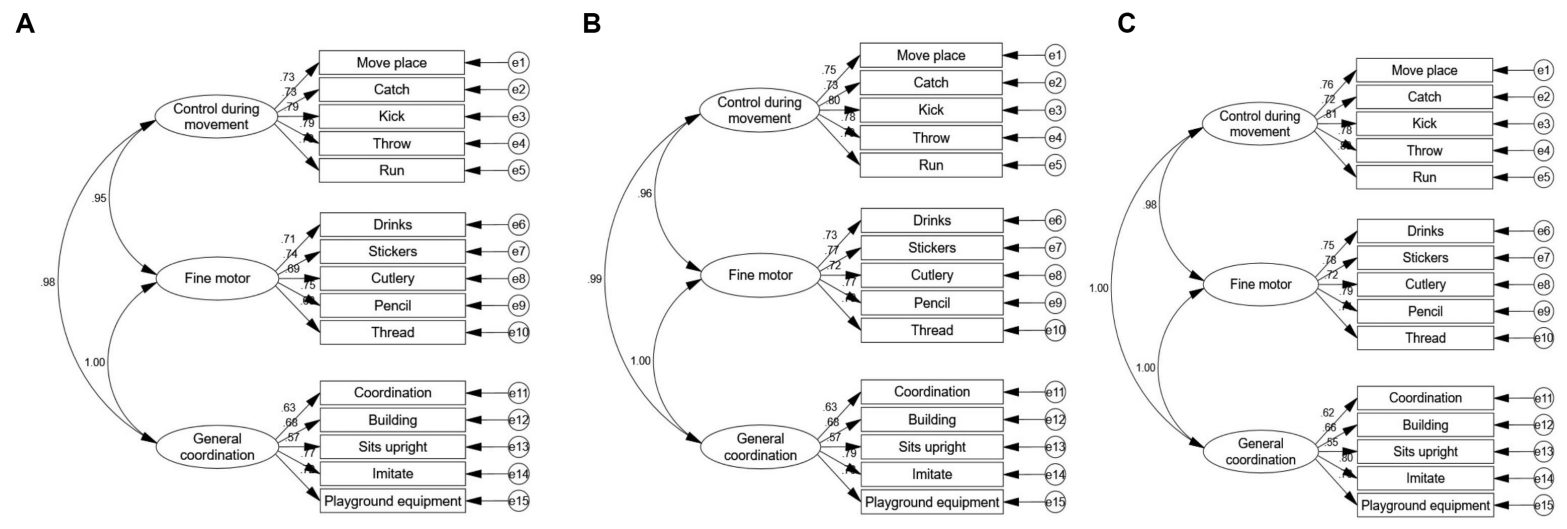
The factor loadings of little LDCDQ-CH using confirmatory factor analysis by age. **(A)** Factor model in children aged 3 years old (n-12849, x/df-106.389, *p* < 0.001, NFI-0.918, CFI-0.918, IFI-0.918, RMSEA 0.091, TL1-0.902). **(B)** Factor model in children aged 4 years old (n-23706, x/df-165.102, *p* < 0.001, NFI-0.928, CFI-0.925, IFI-0.928, RMSEA-0.088, TLI-0.913). **(C)** Factor model in children aged 5 years old (n-17069, x/df-128.674, *p* < 0.001, NFI-0.933, CFI-0.933, IFI-0.933, RMSEA-0.086, TLI-0.919).

In contrast, an CFA based on the original construct of the LDCDQ demonstrated acceptable model fit indices (x^2^/df = 402.731, *p* < 0.001, NFI = 0.927, CFI = 0.927, IFI = 0.927, RMSEA = 0.089), and these values indicate an acceptable fit for the model (NFI, CFI and IFI > 0.9). However, the RMSEA value of 0.089 suggests reasonable errors of approximation within the population, as RMSEA values below 0.05 are desirable. The majority of factor loadings in the 15-item model were statistically significant and above 0.6 (each *p* < 0.001; see Figure 2). This indicates a strong relationship between the observed variables and their respective latent factors. Furthermore, the model fit remained acceptable across different age groups and genders (see Figures 2, 3).

### Concurrent validity

The results indicated moderate correlations between the total score and subscale scores (“Control during movement,” “Fine motor,” and “General coordination”) of the LDCDQ-CH and the total score and sub-scale scores (“Communication,” “Gross motor,” “Fine motor,” “Problem-solving,” “Personal-social”) of the ASQ-3 (each *p* < 0.001, Table 3) within the entire participant sample (*n* = 51,110). Similar associations were observed between the two scales when considering children of different age groups and genders (each *p* < 0.001, Table 3). However, no significant associations were found between the scores of the LDCDQ-CH and the MABC-2 (total score, “Manual dexterity,” “Aiming and catching,” and “Balance”) (*n* = 145) (*p* = 0.153, 0.174, 0.102, and 0.131, respectively).

**Table 3:** Concurrent validity of LDCDQ-CH when compared with ASQ-3 by different age and sex.

LDCDQ	ASQ-3				
	Communication	Gross motor	Fine motor	Problem solving	Personal-social
Age (year old)					
3 (*n* = 12,849)					
Total score	0.458***	0.460**	0.512**	0.483**	0.429**
Control during movement	0.404***	0.453**	0.412**	0.416**	0.360**
Fine motor	0.442***	0.421**	0.539**	0.478**	0.440**
General coordination	0.435***	0.416**	0.477**	0.456**	0.399**
4 (*n* = 21,192)					
Total score	0.411***	0.493**	0.483**	0.407**	0.390**
Control during movement	0.376***	0.477**	0.397**	0.359**	0.340**
Fine motor	0.397***	0.443**	0.503**	0.412**	0.408**
General coordination	0.380**	0.464**	0.455**	0.372**	0.346**
5 (*n* = 17,069)					
Total score	0.443***	0.502**	0.492**	0.414**	0.391**
Control during movement	0.414***	0.486**	0.446**	0.389**	0.364**
Fine motor	0.417***	0.451**	0.484**	0.406**	0.392**
General coordination	0.418***	0.477**	0.457**	0.374**	0.347**
Sex					
Males (*n* = 27,404)					
Total score	0.431***	0.475**	0.499**	0.456**	0.426**
Control during movement	0.390***	0.461**	0.407**	0.398**	0.367**
Fine motor	0.410***	0.427**	0.525**	0.460**	0.446**
General coordination	0.409***	0.446**	0.465**	0.419**	0.379**
Females (*n* = 23,706)					
Total score	0.432***	0.477***	0.512***	0.439***	0.401***
Control during movement	0.405***	0.476***	0.451***	0.395***	0.366***
Fine motor	0.407***	0.414***	0.524***	0.448***	0.406***
General coordination	0.403***	0.450***	0.467***	0.395***	0.357***
Total population (*n* = 51,110)					
Total score	0.431**	0.474**	0.505**	0.448**	0.416**
Control during movement	0.394**	0.465**	0.421**	0.394**	0.361**
Fine motor	0.410**	0.420**	0.528**	0.454**	0.434**
General coordination	0.407**	0.447**	0.468**	0.409**	0.372**

**p < 0.01; ***p < 0.001 (using Pearson correlation analysis).

### Discriminant validity

Significant differences were observed in the total LDCDQ-CH scores among the three groups (motor impairment, at-risk motor impairment, and typical performance as assessed using MABC-2) with statistical significance (each *p* < 0.05). Specifically, when considering age and gender, a significant difference was found between the at-risk motor impairment group and the typical performance group (*p* < 0.05) in children aged 4,5. However, most of these differences were not significant in children aged 3. In the comparison among the three groups (motor impairment, at-risk motor impairment, and typical performance), only the total score of the LDCDQ-CH and general coordination showed significant differences in both male and female children (Table 4).

**Table 4:** Discriminant validity of LDCDQ-CH by different age and sex.

LDCDQ	Significant motor impairment (≤5th centile of MABC-2)	At-risk motor impairment (6 ~ 16th centile of MABC-2)	Typical performance (>16th centile of MABC-2)	*F* value
Age (year old)				
3 (*n* = 31)				
Total score	67.570 (11.487)	60.000 (21.213)	67.910 (9.159)	0.917
Control during movement	23.000 (3.606)	20.000 (7.071)	22.546 (3.113)	0.930
Fine motor	23.286 (3.729)	20.000 (7.071)	22.955 (3.579)	1.049
General coordination	21.286 (4.424)	20.000 (7.071)	22.409 (3.362)	0.712
4 (*n* = 52)				
Total score	50.250 (22.603)*	70.750 (4.924)*	69.360 (6.875)	8.214^##^
Control during movement	18.000 (8.524)*	23.750 (1.893)*	22.909 (2.666)	3.881^#^
Fine motor	15.750 (6.652)*	24.000 (1.155)*	23.636 (2.024)	16.158^###^
General coordination	16.500 (7.594)*	23.000 (2.828)*	22.818 (2.67)	6.135^##^
5 (*n* = 62)				
Total score	63.600 (6.986)*	64.500 (7.594)	72.130 (5.714)	123.164^###^
Control during movement	20.400 (2.966)*	21.750 (3.948)	23.962 (2.738)	58.904^###^
Fine motor	22.800 (2.168)	22.750 (2.63)	24.302 (1.727)	142.65^###^
General coordination	20.400 (2.793)*	20.000 (2.944)	23.868 (1.861)	122.623^###^
Sex				
Males (*n* = 71)				
Total score	56.130 (17.892)*	65.830 (10.998)*	68.510 (8.173)	3.540^#^
Control during movement	18.625 (6.070)*	22.500 (3.987)	22.754 (2.996)	2.865
Fine motor	19.375 (6.278)*	22.667 (3.882)	23.263 (2.882)	2.719
General coordination	18.125 (6.151)*	20.667 (4.274)	22.491 (2.861)	4.228^#^
Females (*n* = 74)				
Total score	67.88 (8.526)	66.500 (9.327)	72.000 (5.329)	3.479^#^
Control during movement	23.25 (2.765)	21.750 (3.948)	23.823 (2.577)	2.340
Fine motor	23.125 (3.044)	22.750 (2.63)	24.307 (1.478)	3.375
General coordination	21.500 (3.207)*	22.000 (2.944)	23.871 (2.036)	4.535^#^
Total population (*n* = 145)				
Total score	62.000 (14.837)*	66.100 (9.814)	70.330 (7.032)	6.060^##^
Control during movement	20.938 (5.144)*	22.200 (3.765)	23.311 (2.825)	3.112^#^
Fine motor	21.250 (5.145)*	22.700 (3.268)	23.807 (2.312)	4.737^#^
General coordination	19.813 (5.049)*	21.200 (3.676)	23.210 (2.551)	8.584^###^

^#^*p* < 0.05, ^##^*p* < 0.01, ^###^*p* < 0.001 (One-way ANOVA). *p < 0.05 [post-hoc comparison with children of typical performance using Fisher’s Least Significant Difference (LSD)].

## Discussion

The current study aimed to translate, adapt and assess the age- and sex-specific usefulness of the LDCDQ in Chinse preschoolers using a large population-based sample. Our findings provide evidence that the LDCDQ-CH demonstrates good to fair reliability and validity across different ages and genders among Chinese preschoolers.

Regarding internal reliability, our results indicated excellent internal consistency for the LDCDQ-CH in Chinese children aged 3–5 years old. The high Cronbach’s alpha values suggest that all items in the LDCDQ-CH effectively measure children’s motor performance. With the exception of the item “Sits upright,” the deletion of any item led to lower item-deleted Cronbach’s alpha values compared to the total value. Furthermore, the split-half coefficient demonstrated that both halves of the data contributed equally to the measurement, indicating high accuracy in assessing motor development using the LDCDQ-CH. These findings support the instrument’s internal consistency and confirm its comparability to the original version and other language versions (Caravale et al., 2014a).

An EFA was conducted to evaluate the construct validity of the LDCDQ-CH. The analysis revealed a one-factor structure, different from the multi-factor construct validity originally reported for the LDCDQ. Conversely, the CFA aligned more closely with the original scale’s three-factor structure. The CFA demonstrated that the 15-item model of the LDCDQ-CH fitted the data acceptably, evidenced by satisfactory factor loadings. This provides further support for the questionnaire’s validity in assessing motor development across different ages and genders. The three-factor structure of the LDCDQ-CH, consisting of general coordination, fine motor skills, and control during movement, aligns with the original version of the LDCDQ (Rihtman et al., 2011). However, it is worth noting that the Canadian version of the Little DCDQ demonstrated a two-factor structure with different item distributions (Wilson et al., 2015), and another study also on Canadian children reported a single-factor structure (Hudson and Willoughby, 2022), differing from the original version. This suggests the need for further exploration to better understand the representation of motor domains by the items in the LDCDQ.

Concurrent validity was demonstrated by the positive and moderate correlation between the LDCDQ-CH and ASQ-3, based on our large population sample (*n* = 51,110). Both instruments address the functional manifestations of motor skills in daily activities as reported by parents. In our study, concurrent validity was also evaluated with a sub-sample of preschoolers (*n* = 145) who underwent objective measurement of motor performance using the MABC-2. Consistent with previous population-based research (Schoemaker et al., 2006), our results indicated weaker but still significant associations between the LDCDQ-CH and the MABC-2 compared to those between the LDCDQ-CH and the ASQ-3. This may suggest that the MABC-2 measures specific motor skills that differ from the parent-reported LDCDQ.

We found no significant variations in the internal reliability, construct validity, and concurrent validity of the LDCDQ-CH across different ages, including an extended age range of up to 5 years and 11 months. These findings suggest that using the LDCDQ up to 5 years old is appropriate for Chinese children. Although sex disparities may exist in nations with differing motor profiles between genders, such as in China (Ke et al., 2020), our study found consistent results regarding reliability and validity of the LDCDQ-CH across both girls and boys. Previous research has shown that there is no gender difference in parental reports of their child’s motor activity, which are strongly associated with the motor competence of the children (Ferreira et al., 2020; Ke et al., 2021). These findings align with and further support our own results.

Further analysis of discriminant validity revealed that the LDCDQ-CH could differentiate between the three groups of at-risk and typical performance for 4- and 5-year-old children, but not for 3-year-old children, which aligns with a previous study conducted in the Dutch population (Cantell et al., 2018). These findings support the recommendations by the European Academy of Childhood Disability (EACD) that a diagnosis of DCD is not recommended before the age of 5 (Blank et al., 2019). Additionally, the discriminant power was nearly insignificant in girls in our study. It may be necessary to adjust for items related to daily activity in girls to enhance the discriminant power of the LDCDQ-CH within specific cultural contexts.

Furthermore, our study found that older children demonstrated higher scores on the LDCDQ-CH, indicating better parent-reported functional skills compared to their younger counterparts. This aligns with previous studies reporting an age-related trend (Rivard et al., 2014; Caravale et al., 2014a), suggesting that children become more capable with increasing age. However, our findings contrast with a report indicating that children’s age did not affect their scores on the LDCDQ (Cantell et al., 2018). Indeed, the instructions of the LDCDQ emphasize that parents should compare their child’s motor performance to that of peers of the same age., and nonetheless, this report did find developmental trends in the correlation coefficients between the LDCDQ and the MABC-2 test across the ages of 3 to 5. This may suggest that older children’s performance is less influenced by variables of motor skill and more reflective of their inherent capabilities.

In our study, parents reported that girls performed a better motor coordination skills than boys, consistent with previous research highlighting that girls tend to outperform boys in motor behaviors (Rivard et al., 2014; Wilson et al., 2015; Cantell et al., 2018; Fu et al., 2022). The results may suggest some items in the LDCDQ could lead to the sex-differences which were reflected by their parents’ observation and judgment. Separate cutoffs for different ages and genders were not generated in the original version of the LDCDQ. As recommended by the EACD consensus, parent reports of motor and coordination difficulties indicate the need for further standardized assessment. The age and sex differences noted in our study suggest that future research should consider specific norms for the LDCDQ-CH based on age and gender.

## Conclusion

In conclusion, our study provides valuable insights into the age- and sex-specific applicability of the LDCDQ-CH as a tool for assessing motor development in Chinese preschoolers. The findings demonstrate that the LDCDQ-CH exhibits good reliability and validity across different ages and genders, supporting its use as a reliable instrument in the Chinese context. Our study further confirms the suitability of extending the LDCDQ-CH usage up to 5 years and 11 months in Chinese children. These findings contribute to the understanding of motor development assessment and provide important insights for early identification and intervention in motor impairments. Future research should consider establishing specific norms by age and sex to enhance the applicability of the LDCDQ-CH in diverse populations. Overall, the LDCDQ-CH proves to be a valuable tool for evaluating motor development in Chinese preschoolers, enabling early identification and support for children at risk of motor impairments.

## Data availability statement

The raw data supporting the conclusions of this article will be made available by the authors, without undue reservation.

## Ethics statement

The studies involving humans were approved by the Ethics Committee of Shanghai First Maternity and Infant Hospital (KS18156). The studies were conducted in accordance with the local legislation and institutional requirements. Written informed consent for participation in this study was provided by the participants’ legal guardians/next of kin.

## Author contributions

JH: Conceptualization, Funding acquisition, Investigation, Methodology, Resources, Supervision, Writing – original draft, Writing – review & editing. TR: Writing – original draft, Writing – review & editing. YP: Data curation, Formal analysis, Investigation, Methodology, Project administration, Resources, Writing – original draft. TW: Data curation, Formal analysis, Project administration, Writing – original draft. YS: Data curation, Formal analysis, Project administration, Writing – original draft. WD: Conceptualization, Investigation, Methodology, Supervision, Writing – original draft, Writing – review & editing.
